# A study of cochlear and auditory pathways in patients with tension-type headache

**DOI:** 10.1186/s10194-015-0557-x

**Published:** 2015-08-13

**Authors:** Hang Shen, Wenyang Hao, Libo Li, Daofeng Ni, Liying Cui, Yingying Shang

**Affiliations:** Department of Neurology, Peking Union Medical College Hospital, Chinese Academy of Medical Sciences and Peking Union Medical College, Beijing, China; Department of Otorhinolaryngology, Peking Union Medical College Hospital, Chinese Academy of Medical Sciences and Peking Union Medical College, Beijing, China

**Keywords:** Tension-type headache, Otoacoustic emissions, Acoustic reflex, Phonophobia, Brainstem

## Abstract

**Background:**

The purpose of this study was to systematically evaluate the function of cochlear and auditory pathways in patients suffering from tension-type headache (TTH) using various audiological methods.

**Methods:**

Twenty-three TTH patients (46 ears) and 26 healthy controls (52 ears) were included, and routine diagnostic audiometry, extended high-frequency audiometry, acoustic reflex (ASR), transient evoked otoacoustic emissions (TEOAEs), distortion product otoacoustic emissions (DPOAEs) and suppression TEOAEs were tested.

**Results:**

The TTH group showed higher thresholds (*P* < 0.05) for both pure tone and extended high-frequency audiometry at all frequencies except for 9, 14 and 16 kHz. All ASR thresholds were significantly higher (*P* < 0.05) in the TTH group compared with the controls, except for the ipsilateral reflex at 1 kHz, but the threshold differences between the ASR and the corresponding pure tone audiometry did not differ (*P >* 0.05). For the DPOAEs, the detected rates were lower (*P* < 0.05) in the TTH group compared with the controls at 4 and 6 kHz, and the amplitudes and signal to noise ratio (S/N) were not significantly different between groups. No differences in the TEOAEs (*P* > 0.05) were observed for the detected rates, amplitudes, S/Ns or contralateral suppression, except for the S/Ns of the 0.5-1 kHz TEOAE responses, which were significantly higher (*P* < 0.05) in the TTH group.

**Conclusions:**

The results of our study indicate that subclinical changes in cochlear function are associated with TTH.

## Background

Tension-type headache (TTH) is the most prevalent type of primary headache, and its lifetime prevalence in the general population ranges from 30 to 78 % [1]. TTH is characterized by episodes of headache lasting minutes to days, with mild to moderate pain that is typically bilateral, pressing or tightening in quality. TTH pathophysiology is complex and incompletely understood. Neuron sensitization in the central nervous system is regarded as one of the major mechanisms underlying this condition [2, 3].

Phonophobia is regarded as the most common auditory symptom in patients with migraines. However, phonophobia can also occur in TTH patients at relatively high rates of 38–40.9 % [4, 5], although the frequency and intensity are not as serious as in migraineurs. Nevertheless, noise can be an important trigger and aggravating factor in both TTH and migraine patients [4, 6, 7]. A study by Spierings et al. [6] showed that noise is a precipitating and aggravating factor in 29 % and 65 % of TTH patients, respectively. This finding led to speculation that these primary headaches may be associated with dysfunction of the auditory system. Several studies [8–10] examining the auditory system using auditory brainstem responses and otoacoustic emissions (OAEs) have been conducted in migraineurs, and the results suggest that subclinical dysfunctions of the cochlear and auditory pathways are related to migraines. Because phonophobia and noise are also related to TTH as complicating symptoms and participating or aggravating factors, we investigated whether these types of dysfunctions also exist in TTH patients. Furthermore, studies have shown that cochlear-vestibular symptoms, such as tinnitus and vertigo, are also observed in TTH patients, although at lower prevalences than in migraine patients [11, 12]. This finding is also suggestive of potential relationships between TTH and the auditory system. However, to our knowledge, no specific studies exploring the auditory system have been performed in TTH patients.

Pure tone audiometry (PTA) is the most common hearing test used to obtain hearing threshold levels in individuals for frequencies ranging from 0.25–8 kHz, enabling the identification of the type, degree and configuration of hearing loss. Extended high-frequency audiometry evaluates hearing thresholds for frequencies higher than 8 kHz and has been suggested as a method for monitoring the effects of noise exposure, ototoxic medication, and hearing loss resulting from other causes [13–17]. High frequencies appear to be more susceptible to external factors, such as medication and noise, than the middle and low frequencies [15, 17]. Although audiometry cannot clarify the lesion level of auditory dysfunction, it remains a useful tool for evaluating the integral function of the auditory system.

OAEs were first reported by Kemp in 1978 [18]. OAEs are sounds that arise in the cochlea and move through the middle ear and straight into the ear canal, where they can be detected using sensitive equipment. OAEs are thought to be the byproducts of the preneural mechanisms of the cochlear amplifier and are related to the normal function of the outer hair cells (OHCs). Transient evoked otoacoustic emissions (TEOAEs) and distortion product otoacoustic emissions (DPOAEs) are the two most common types of evoked otoacoustic emissions (EOAEs). For TEOAEs, a “click” stimulus is provided to the ear to evoke broad-spectrum responses of the highest intensity in the mid-frequency spectrum, 1–4 kHz. DPOAEs are the product of an intermodulation distortion generated by the cochlea responding to the stimulation of two simultaneous pure tones. The frequencies of these two stimuli are close to each other, and they are described as primary tones. The response is a tonal signal that does not exist in the eliciting stimuli; therefore, it is called a “distortion product”. Because EOAE testing can detect fine changes in the OHCs that are undetectable by other methods, it permits a sensitive evaluation of cochlear function [14, 17, 19] and objectively monitors dynamic changes in the cochlea before any functional and significant hearing loss appears [20].

OAE suppression testing is a technique that assesses the brainstem auditory reflex, which is a reduction in OAE amplitude when stimulation occurs in the contralateral ear [21]. The reflex arc includes the auditory nerve, cochlear nucleus, trapezoid body, superior olivary complex, olivocochlear bundle, inferior vestibular nerve, outer hair cells and inner hair cells. Because contralateral suppression is mediated by the efferents from the medial superior olivary complex to the OHCs, it is a useful tool for studying the cochlear efferents in the brainstem.

The acoustic reflex (ASR) refers to the reflexive contraction of the middle ear muscles caused by loud sound stimulation. The acoustic reflex threshold (ART) is the lowest level of a sound stimulus that can elicit an ASR response, i.e., a measurable change in acoustic emittance. The ASR arc is composed of the cochlea, auditory nerve, ventral cochlear nucleus, trapezoid body, superior olivary complex, facial nucleus and facial nerve. Previous studies have shown that ARTs are related to thresholds of the uncomfortable loudness level (UCL) and can be used to estimate the UCL [22–24]. In previous studies, significantly lower thresholds were observed in subjects with hypersensitivity to sound caused by various diseases [25, 26].

The purpose of this study was to systematically evaluate the function of the cochlear and brainstem auditory pathways in patients suffering from TTH using pure tone audiometry, extended high-frequency audiometry, the ASR, TEOAEs, DPOAEs and TEOAE suppression to examine the potential relationship between TTH and the auditory system.

## Methods

### Subjects

The TTH group included patients recruited from the Headache Clinic of the Neurology Outpatient Department at the Peking Union Medical College Hospital. Twenty-three patients with TTH (7 females and 16 males) were involved in this study, and 46 ears were tested. Of the 23 patients, 16 had frequent episodic TTH, and 7 had chronic TTH. Patients with episodic TTH were studied during attack-free periods. The mean patient age was 34 ± 9 years (range 18–52). TTH was diagnosed according to the criteria of the International Headache Society. The diagnosis was consistent with the International Classification of Headache Disorders-3 (beta version) codes 2.1, 2.2 and 2.3 [1]. Patients with 2.4 probable TTH were excluded. No patients had any neurotologic symptom such as tinnitus, hearing loss, dizziness or vertigo. No history of chronic otological disease, ear surgery, noise exposure, ototoxicity, or any systemic metabolic or autoimmune disease associated with hearing loss was reported, and no history of central nervous system disease or other primary headache disorders, except for TTH, was reported.

The healthy controls were volunteers recruited from the hospital staff of Peking Union Medical College Hospital. Twenty-six healthy controls (52 ears, 9 females and 17 males) were included, with a mean age of 35 ± 8 years (range 24–51). No healthy controls reported any primary or secondary headache, any history of ear surgery, chronic otological disease, noise exposure, ototoxicity, central nervous system disease or any systemic, metabolic or autoimmune diseases associated with hearing loss. The study protocol was approved by the Peking Union Medical College Hospital Institutional Review Board in accordance with the Declaration of Helsinki. Informed consent was obtained from all subjects.

### Audiometry

All of the subjects underwent an otoscopic examination performed by an ENT doctor, and those subjects with any type of external ear or middle ear disease were excluded. Next, the subjects underwent tympanometry (Madsen Otoflex 100 and Otodiagnostic Suite immittance meter, GN, Denmark). Subjects with any type of tympanometric disorder were excluded. Pure tone audiometry was performed using a Conera audiometer (Madsen, GN, Denmark) (ISO 389). First, frequencies from 0.25 to 8 kHz were tested using TDH 39 headphones with a step size of 5 dB (ISO 8253–1:1989). The extended high frequencies (9, 10, 11.2, 12.5, 14 and 16 kHz) were tested with Sennheiser HDA-200 headphones using the above-described method. All equipment was calibrated every 12 months.

### ART testing

Ipsilateral and contralateral ARTs were measured using an Otoflex 100 and Otodiagnostic Suite immittance meter (Madsen, GN, Denmark). The ASR responses to four different stimuli (0.5-, 1-, 2- and 4- kHz tones) were recorded at a probe tone of 226 Hz. An auto threshold search program was applied in a combined descending-ascending manner, and an ASR response was detected when an admittance change exceeded 0.04 mmho. To begin, a stimulus was presented at an intensity of 70 dB HL, and its intensity level was decreased or increased in 5-dB steps according to the responses. The maximum output intensity for all tones was 105 dB HL.

### Otoacoustic emission testing

TEOAEs and DPOAEs were evaluated with an otoacoustic emission analyzer (Madsen, CELESTA 503, Denmark) in a soundproof booth.

For the DPOAE testing, two pure tone stimuli were presented simultaneously to the ear canal at different frequencies, and the distortion-product component at 2f1-f2 was recorded. The DPOAE response amplitude was measured as a function of f2 frequency, with the f2/f1 ratio fixed at 1.2 at a fixed stimulus level (L1 = 65 dB SPL, L2 = 55 dB SPL). Eight pairs of stimuli were presented with an f2 frequency at 0.75, 1, 1.5, 2, 3, 4, 6 or 8 kHz. For each frequency, the DPOAE signal was considered detectable if the signal to noise ratio (S/N) exceeded 3 dB at 2f1-f2.

The TEOAEs were obtained at 0.5-4 kHz with a stimulation of 40-μsec clicks and a linear protocol. The stimulus level in the external ear canal was 80 ± 3 dB SPL. The click rate was 50 Hz, and the time window for analysis was 6–18 ms post-stimulus. In total, 1000 sweeps were averaged. A TEOAE response was regarded detectable if the S/N exceeded 3 dB. If the reproducibility percentages exceeded 70 % and the stimulus stability was higher than 80 %, the response was considered acceptable for analysis. For TEOAE suppression testing, white noise was generated by an ORBITER 922 audiometer (Madsen, Denmark) and was presented to the contralateral ear through a TDH 39 headphone. The intensity of white noise was fixed at a 40-dB sensation level to achieve the optimal measurement parameters [21].

### Statistical analysis

All statistical analyses were performed using SPSS for Windows, version 17.0. The threshold, amplitude and S/N results for the audiometry, the ASR, DPOAEs and TEOAEs were compared in the TTH patients and controls using the independent samples *t*-test. The detected rates of ASR, DPOAEs and TEOAEs were compared between the TTH patients and controls using the *χ*^2^ test. To determine whether the TEOAE amplitude changes with and without contralateral noise, paired *t*-tests were used, and differences in the extent of suppression between the patients and controls were compared using the Mann–Whitney *U* test.

## Results

The demographic and clinical characteristics of the study groups are shown in Table 1. No differences in gender or age were detected between the TTH patients and controls.

**Table 1:** Demographic and clinical characteristics of the studied groups

Demographic and clinical characteristics	TTH patients (*n* =23)	Controls (*n* = 26)
Male/female	16/7	17/9
Age, years	18-52 (34.17 ± 9.15)	24-51 (34.88 ± 7.88)
Type of TTH		-
Infrequent episodic TTH	0	
Frequent episodic TTH	16 (69.6 %)	
Chronic TTH	7 (30.4 %)	
Months from the onset of TTH	Mean 79; SD 87; Range 3-360	
Auditory-related symptoms		
Phonophobia as premonitory symptom	6 (26.1 %)	-
Phonophobia as complicating symptom	8 (34.8 %)	
Noise as trigger	7 (20.4 %)	

**Fig 1:**
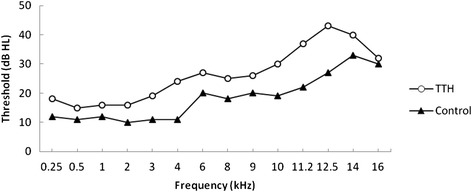
Comparison of pure tone and extended high-frequency hearing thresholds between the two groups (mean)

The TTH group had higher thresholds in both pure tone and extended high-frequency audiometry (Fig. 1), with significant differences observed at all frequencies except for 9, 14 and 16 kHz.

The ipsilateral and contralateral ARTs were obtained in most subjects, and except for the ipsilateral reflex at 1 kHz, all thresholds were significantly higher in the TTH group compared with the control group (Table 2). However, the differences in threshold values between the ASR and PTA did not differ between the groups.

**Table 2:** Acoustic reflex thresholds results

		Number of cases without reflex		Thresholds (mean ± SD)		Reflex threshold – pure tone threshold	
		(*n* (%))		(dB HL)		(dB)	
Frequencies (kHz)		TTH	Controls	TTH	Controls	TTH	Controls
Ipsilateral	0.5	0	0	83 ± 12^*^	75 ± 12^*^	68 ± 15	64 ± 13
	1	1 (2.2 %)	0	85 ± 10	83 ± 12	70 ± 15	71 ± 12
Contralateral	2	1 (2.2 %)	0	84 ± 9^*^	79 ± 10^*^	68 ± 14	68 ± 10
	4	1 (2.2 %)	0	84 ± 11^*^	76 ± 12^*^	60 ± 20	65 ± 11
	0.5	4 (8.7 %)	2 (3.8 %)	92 ± 9^*^	86 ± 10^*^	—	—
	1	3 (6.5 %)	0	91 ± 8^*^	86 ± 11^*^	—	—
	2	2 (4.3 %)	0	91 ± 8^*^	86 ± 10^*^	—	—
	4	2 (4.3 %)	1 (1.9 %)	92 ± 9^*^	86 ± 10^*^	—	—

*P-value <0.05

DPOAEs were detected at different rates in the two groups. The DPOAE rates and values are presented in Table 3. The observed rates were lower at most frequencies in the TTH group, with significant differences at 4 and 6 kHz; however, the amplitudes and S/Ns were not significantly different between groups. No significant differences were observed in the detected TEOAE rates, amplitudes or S/Ns, except that the S/N was higher in the TTH group at frequencies between 0.5 and 1 kHz (Table 4). For contralateral TEOAE suppression, significant suppression occurred in both groups, which was confirmed by a paired *t*-test to assess the TEOAEs in silence and with noise (Table 5). However, the Mann–Whitney *U* test showed no difference in the mean suppression value between groups.

**Table 3:** DPOAE results

Frequency (kHz)	Detected rate (n (%))			Amplitude (dB SPL)			S/N (dB)		
	TTH	Control	*P*-value	TTH	Control	*P*-value	TTH	Control	*P*-value
0.75	43 (93.5)	45 (86.5)	*0.257*	3.6 ± 6.8	2.3 ± 6.5	*0.354*	14.9 ± 7.8	12.2 ± 8.4	*0.103*
1	42 (91.3)	49 (94.2)	*0.575*	3.6 ± 8.7	3.7 ± 7.3	*0.967*	18.9 ± 9.4	18.6 ± 7.7	*0.867*
1.5	42 (91.3)	50 (96.2)	*0.318*	−0.6 ± 9.6	−1.1 ± 8.0	*0.751*	17.4 ± 10.1	16.4 ± 8.5	*0.583*
2	40 (87.0)	47 (90.4)	*0.592*	−6.7 ± 10.3	−6.5 ± 7.8	*0.929*	15.1 ± 10.7	14.1 ± 7.9	*0.614*
3	31 (67.4)	43 (82.7)	*0.079*	−17.8 ± 10.9	−15.3 ± 7.3	*0.189*	9.3 ± 10.3	11.4 ± 7.5	*0.249*
4	30 (65.2)	47 (90.4)	*0.002*	−11.9 ± 11.0	−10.1 ± 6.8	*0.342*	11.6 ± 11.2	13.2 ± 6.8	*0.409*
6	34 (73.9)	44 (89.8)	*0.044*	−5.8 ± 11.7	−6.8 ± 8.1	*0.614*	12.4 ± 11.9	10.8 ± 8.1	*0.443*
8	30 (65.2)	29 (55.8)	*0.340*	−7.6 ± 11.9	−11.4 ± 9.9	*0.088*	8.2 ± 11.7	3.9 ± 9.9	*0.051*

**Table 4:** TEOAE results

Frequency	Detected rate (n (%))			Amplitude (dB SPL)			S/N (dB)		
	TTH	Control	*P*-value	TTH	Control	*P*-value	TTH	Control	*P*-value
Overall echo level	45 (97.8)	51 (98.1)	0.930	12.7 ± 4.1	12.5 ± 3.5	*0.796*	16.6 ± 4.6	14.9 ± 4.9	*0.059*
0.5-1 kHz	45 (97.8)	51 (98.1)	0.930	11.2 ± 4.3	10.4 ± 3.7	*0.339*	17.2 ± 5.8	14.5 ± 5.1	*0.015*
1-2 kHz	46 (100)	52 (100)	-	5.3 ± 5.9	7.0 ± 4.8	*0.135*	16.9 ± 5.7	17.2 ± 5.3	*0.792*
2-4 kHz	17 (37.0)	15 (28.8)	0.393	−9.5 ± 4.6	−10.1 ± 3.3	*0.437*	3.1 ± 5.0	2.7 ± 3.7	*0.621*

**Table 5:** Otoacoustic emission suppression results

	TTH	Control
OAE_q_ – OAE_n_ (mean ± SD) (dB)	0.97 ± 1.21	0.57 ± 1.57
Paired *t-*test *P* values	*P* = 0.000	*P* = 0.013
TTH vs. Controls *Mann–Whitney U*	*P* = 0.062	

*OAE*_*q*_ TEOAE in quiet, *OAE*_*n*_, TEOAE in noise, SD standard deviation

## Discussion

In the present study, despite the fact that subjects with complaints of hearing loss, tinnitus, vertigo or dizziness were excluded from both groups to rule out the influences of complicating ear diseases, statistically significant differences were observed between the TTH patients and controls in pure tone and high-frequency hearing thresholds (Fig. 1). This finding suggests a subclinical dysfunction of the auditory system in TTH patients. Furthermore, higher cochlear dysfunction risks were indicated by the lower detected DPOAE rates at 4 and 6 kHz in the TTH patients, which could explain the audiometry results, indicating that cochlear dysfunction may contribute to threshold elevation. The higher S/Ns in the TEOAE test at frequencies of 0.5–1 kHz in the TTH group seem to contradict the DPOAE findings. However, the possibility that the higher S/N values may result from lower background noise, not a higher response, makes the S/N value a less than optimal parameter to evaluate cochlear function. Indeed, the S/N is more often used as a criterion to decide whether the response exists. In previous studies, OAEs have been tested in migraineurs, and an amplitude decrease has also been implicated in subclinical cochlear dysfunction [8–10]. However, because the mechanisms of TTH and migraine are different, the causes of cochlear dysfunction may also be different.

Contralateral suppression TEOAEs were evaluated in cochlear efferents in the brainstem, but no significant differences were observed between groups. ART is another test that is related to the auditory pathway at the brainstem level. The results indicate that although the ARTs were higher in the TTH group, the ART and PTA thresholds were not higher than those of the control group. Given the audiometry results, the elevation in the ARTs in the TTH group was likely a result of elevated hearing thresholds; moreover, no ART decrease related to hypersensitivity was detected in the TTH patients. However, in the present study, all hearing tests were performed in interictal states, except for those administered to subjects with chronic TTH, but most patients experienced phonophobia only during attacks. Further studies during headache attacks are required and may still display positive results.

In the present study, cochlear dysfunction was observed in the TTH patients; however, the mechanism of cochlear dysfunction remains unclear. In addition, knowledge about the physiological mechanisms underlying TTH is limited. At present, both peripheral and central mechanisms are thought to play important roles in TTH [2, 3]. Enhanced tenderness to palpation of pericranial myofascial tissues has been reported as the most obvious abnormality in TTH patients [27, 28], and impulses of nociceptive receptors in the pericranial muscles are thought to be transmitted to the brain and perceived as a headache. Myofascial tissue could also be one of the key factors in TTH [29, 30]. Central sensitization is regarded as the most likely mechanism of TTH [2, 3]. Pain sensitivity may occur at both the supraspinal level and the spinal dorsal horn/trigeminal nucleus level. More studies are required to assess whether any similar peripheral changes occur in the auditory system concurrent with the alterations of the somatosensory system.

Another possible explanation for the cochlear dysfunction observed in TTH patients is the ototoxicity of painkillers. Although their ototoxic effects are not as strong as those of other ototoxic medications, such as aminoglycosides and chemotherapeutics, analgesics, including aspirin, non-steroidal anti-inflammatory drugs (NSAIDs), and acetaminophen, do induce ototoxicity [31]. Moreover, among over-the-counter drugs, analgesics are the most widely used in individuals’ daily lives. Patients with frequent episodic and chronic TTH who were included in the present study could have consumed far more NSAIDs, aspirin and acetaminophen as painkillers compared with the control group. This phenomenon may therefore have led to the observed cochlear dysfunction. However, no detailed investigation related to the dose, frequency or duration of analgesic use was conducted here, and further studies are required to clarify this association. In an audiological study of migraines, subclinical dysfunction was also evidenced by decreased DPOAE amplitudes, but no threshold differences were detected [8, 10]. Because patients with migraines also have moderate to severe headaches, the amount of analgesics that they use could be significantly greater than that of controls; thus, the effect of ototoxicity cannot be ignored. However, no threshold elevation has been detected in patients with migraines [8, 10], which suggests that the threshold elevations detected in the present study could be characteristic of TTH itself rather than the ototoxic effects of analgesics.

Circulatory issues also represent a possible cause of cochlear dysfunction that should be taken into consideration, as the cochlea is very sensitive to ischemia and hypoxia, and many functional disabilities or diseases of the inner ear, such as noise-induced hearing loss, endolymphatic hydrops and presbycusis, have been explained by alterations in cochlear blood flow [32]. However, no studies have directly demonstrated that TTH is associated with circulatory changes or ischemia. Studies have shown that glyceryl trinitrate, a pro-drug for nitric oxide (NO), can induce an immediate headache in chronic TTH patients, stronger than in healthy controls, as well as a delayed headache of the tension type, indicating that NO is likely to play a crucial role in TTH [33]. Furthermore, studies in various animal models have shown that increased NO production might be responsible for human hearing disorders such as sudden idiopathic hearing loss, acute noise trauma, presbycusis and other forms of hearing loss [32]. The blood vessel system is one of three NO-dependent regulatory systems within the cochlea. Thus, it is possible that circulatory changes induced the cochlear dysfunction discovered in our study and that NO might be a key trigger [32]. NO also affects the gap junction system and the synaptic signal transfer process, which could also be responsible for the cochlear dysfunction found in TTH patients [32]. Further study in this area is needed.

Phonophobia refers to the fear of loud sounds. People with phonophobia feel uncomfortable with sounds that do not bother others. The mechanism of phonophobia is not well understood, particularly in patients with TTH. Phonophobia is one of the most common symptoms of migraine and can be part of the migraine diagnosis [1, 5]. Studies of contralateral TEOAE suppression in migraineurs have shown an absence of or decrease in suppression, and auditory sensory dysmodulation could exist in migraine patients, with a disturbance in contralateral suppression representing one of the mechanisms associated with phonophobia in these patients [8, 9]. Although less frequent and intense than in migraine, phonophobia is also a common symptom in TTH patients [4, 5]. However, the physiological mechanisms giving rise to TTH differ from those of migraine. Moreover, according to the present study, no significant changes in contralateral suppression were detected in the TTH patients. The different results for TEOAE suppression between patients with TTH and migraine may reflect differences in the underlying mechanisms of these two diseases. In our study, we conducted a thorough evaluation of the auditory system in TTH patients but did not specifically assess the symptom of phonophobia. Thus, although our findings in the auditory system may be related to auditory symptoms such as phonophobia, further research, particularly specifically designed evaluations of phonophobia, is needed to clarify the association between these conditions.

## Conclusions

This study revealed that patients with TTH have subclinical cochlear dysfunction. Given the unclear physical and physiological mechanisms of cochlear dysfunction in TTH, determining auditory function, particularly cochlear function, in TTH patients should remain an active field of research. Whether assessment of cochlear function can help to determine the prognosis of patients with TTH or other types of headaches remains to be determined.

## Abbreviations

TTH: Tension-type headache
PTA: Pure tone audiometry
ASR: Acoustic reflex
ART: Acoustic reflex threshold
OAE: Otoacoustic emission
TEOAE: Transient evoked otoacoustic emission
DPOAE: Distortion product otoacoustic emission
UCL: Uncomfortable loudness level
S/N: Signal to noise ratio
NSAID: Non-steroidal anti-inflammatory drug
NO: Nitric oxide
